# Expression of cancer stem cell biomarkers as a tool for a correct therapeutic approach to hepatocellular carcinoma

**DOI:** 10.18632/oncoscience.163

**Published:** 2015-05-15

**Authors:** Maurizio Romano, Francesco De Francesco, Giuseppe Pirozzi, Enrico Gringeri, Riccardo Boetto, Marina Di Domenico, Barbara Zavan, Giuseppe A. Ferraro, Umberto Cillo

**Affiliations:** ^1^ Department of Surgery, Oncology and Gastroenterology, Hepatobiliary Surgery and Liver Transplantation, Padua University Hospital, Padua (Italy); ^2^ Multidisciplinary department of Medical-Surgical and Dental Specialties, Second University of Naples, Naples (Italy); ^3^ Department of Experimental Oncology, National Cancer Institute, G.Pascale, Naples (Italy); ^4^ Department of Biochemistry, Biophysics and General Pathology, Second University of Naples, Naples (Italy); ^5^ Department of Biomedical Sciences, University of Padua, Padua (Italy)

**Keywords:** biomarkers, hepatocellular carcinoma, early diagnosis, clinic-pathological, prognosis

## Abstract

Liver cancer is the fifth most commonly diagnosed malignancy and the second most frequent cause of cancer death in men worldwide. Amongst liver cancers, hepatocellular carcinoma (HCC) represents the major histological subtype and it is one of the most common malignant human tumors worldwide. Research into the molecular biology of hepatocarcinogenesis has identified several biomarkers, which could provide additional informations in order to better understand the biology of HCC. A large number of biomarkers have been shown to have potential predictive significance and a wide variety of molecular markers have been proven to be excellent diagnostic tools for HCC but it is difficult to characterize HCC with a single biomarker. Thus, signatures of a combination of biomarkers may be more valuable for the diagnosis, staging and prognosis of HCC. Specifically, a correlation of HCC-CSCs phenotype to specific hepatic cancer subtypes and to specific clinical and pathological features has not yet been reported in human liver tumors. In this view we will first discuss the possible sources of liver stem cells and their relation with liver cancer development and we will secondly focus on the prognostic significance of clinical and pathological features of HCC.

## INTRODUCTION

**Figure 1:**
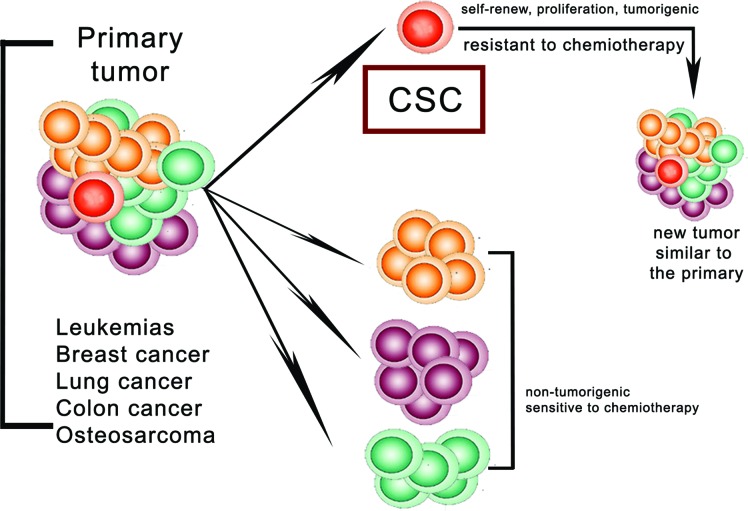
The cancer stem cell hypothesis (adapted from Lobo et al, 2007) Tumors are not viewed as homogeneous masses of proliferating cells but as heterogeneous aberrant tissues containing a hierarchy of cells that originate from a single cancer stem cell (CSC). The CSC hypothesis postulates that a small subpopulation of cancer cells drives tumor growth and metastasis. CSCs are rare, quiescent, and capable of self-renewing and maintaining tumor growth and heterogeneity. Moreover, CSCs share more resistance to therapies, due to antiapoptotic activity and drug resistance (increased levels of drug efflux pumps and multi-drug resistance).

**Figure 2:**
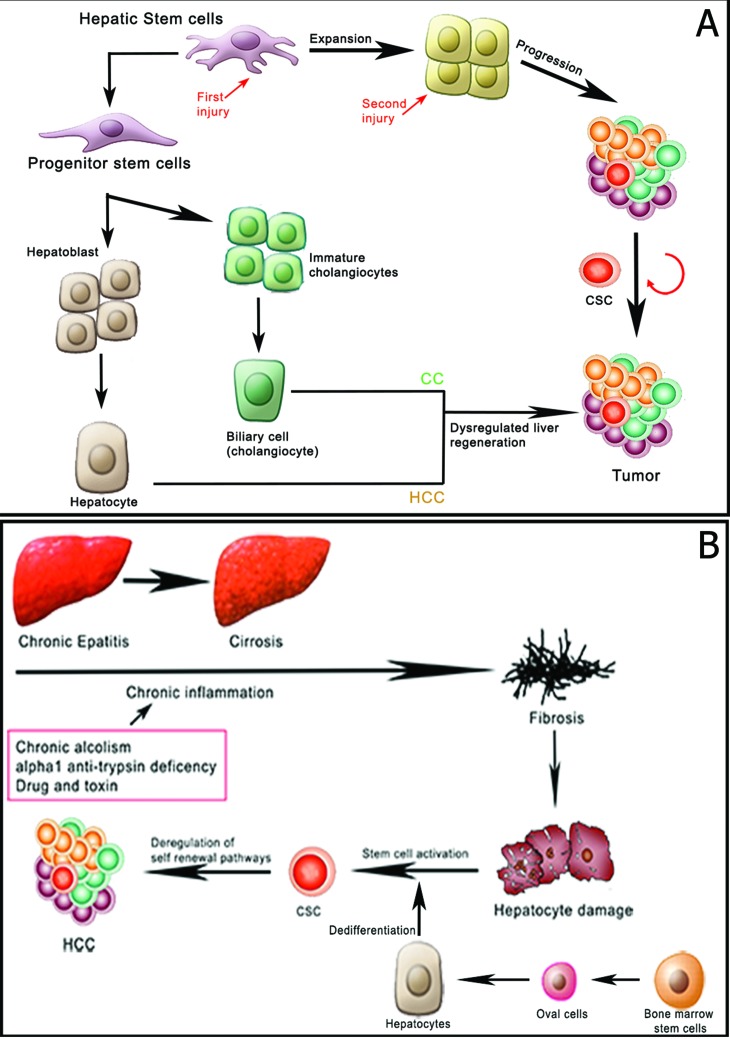
Hepatic stem cells in liver regeneration and HCC (adapted from Yin C et al, 2013; Carpino G et al, 2012) (**A**) Normal cellular hierarchy comprising hepatic stem cells that progressively generate more restricted progenitor cells, yielding all the mature cell types that constitute the liver. Although the some hepatic stem cells have also been proposed to contribute to HCC development, potentially through dysregulation of some aspects of liver regeneration. The accumulation of further epigenetic mutations during neoplastic progression may result in the emergence of CSC, which propagates the tumor. (**B**) Liver fibrosis is the generic response to chronic liver injury. Most evidence suggests that fibrosis promotes HCC by involvement of inflammatory cells, integrin signaling, growth factor interactions with the ECM and communication between activated hepatic stem cells and tumor cells. In this case, hepatic stem cells (present between endothelial cells and cancer cell trabeculae in HCC patients) increases proliferation and migration of human HCC cells.

Liver cancer is the fifth most commonly diagnosed tumor and the second most frequent cause of cancer death in men worldwide. Amongst liver cancers, hepatocellular carcinoma (HCC) represents the major histological subtype and it is one of the most common malignant human tumors [1]. Intrahepatic cholangiocarcinoma (ICC) is the second most frequent type of liver cancer. Although considerable efforts that have been made in the treatment of HCC such as surgical resection, liver transplantation or and chemotherapy, the mortality rate remains high, and it is largely due to relapse after surgery or formation of intra-hepatic metastases [2]. Combined HCC-cholangiocellular carcinoma (HCC-CCA), a histologycal subtype showing both hepatocellular and biliary epithelial differentiation, has been reported [3]. Indeed, recent immunohistochemical studies of stem cell markers suggest that HCC, and HCC-CCA are histologically heterogeneous and contain a subset of cells expressing a variety of stem cell markers [4]. Cancer stem cells (CSCs) have been defined as “a cell within a tumor that possesses the capacity to self-renew and to cause the heterogeneous lineages of cancer cells that comprise the tumor” [5]. The CSCs hypothesis postulates that a small subpopulation of cancer cells drives tumor origin, growth and metastasis. According to this hypothesis, tumors are organized into a hierarchy of heterogeneous cell populations and only a small subset of cells has the ability to drive and sustain tumor growth [6] (Figure 1). CSCs are endowed with stem cells properties, therefore they are capable of extensive proliferation, self-renewal and ability to differentiate into non tumorigenic cancer cells. CSCs have been first identified and isolated in human acute myeloid leukemia [7] as a subpopulation of cells that expressed the CD34 surface marker, but lacked the CD38 marker. The properties of CSCs in solid tumors remained relatively undefined until 2003, when their existence was first reported in human breast cancer tumors [8]. A series of breast CSCs' surface markers were characterized and all of them have been tested and verified. For instance, Camerlingo and colleagues [9] showed that the CD44+/CD24-/low phenotype is correlated with the more aggressive clinical and pathological features of the disease. More recently, highly tumorigenic cells with different surface marker phenotypes were identified. For example, CD133 (prominin-1), an apical plasma membrane protein predominantly on embryonic epithelial structures, has been reported to be an important CSC marker in a number of solid malignancies, including brain tumor [10], prostate carcinoma [11], ovarian carcinoma [12], colorectal cancer [13], osteosarcoma [14] and lung cancer [15]. Besides breast cancer, CD44 is also involved in the tumorigenic CSCs of colon cancer, ovarian carcinoma, head and neck squamous cell carcinoma [16] and prostate cancer. In liver cancer, the sickness generally develops in the setting of chronic liver disease, in which continuous inflammation and hepatocyte regeneration occur. These processes, common to other tumor subtypes [17], include also the expansion of stem/progenitor cells, accumulation of genetic and/or epigenetic mutations, and alterations of the microenvironment (Figure 2A, B). Hepatic stem cells are markedly elevated in chronic liver disease [18]. In this context, the hepatocyte's proliferative capacity is considered virtually infinite. Activation of stromal cells may induce various signaling pathways, including cytokines such as Wnt, FGF, PDGF, VEGF and TGF-beta and promote the development of liver CSCs. Hepatic stem/progenitor cells derive from the canals of Hering (also named “liver stem cell niche”), bile canaliculi lined with hepatocytes and cholangiocytes [19]. In HCC, CSC markers include epithelial cell adhesion molecule (EpCAM), CD133, CD90, CD44, CD24, CD13 and oval cell marker OV6, as well as Hoechst dye efflux or aldehyde dehydrogenase activities, some of which may functionally support CSC phenotypes, including highly invasive features and chemoresistance [20]. CD133, a hematopoietic stem cell marker, was used to isolate stem-like cells from HCC cell lines [21]. CD133+ cells possessed some stem cell properties, including higher proliferative potential, greater colony-forming efficiency, self-renewal and differentiating capacity and, additionally, they could initiate tumor growth in vivo. Furthermore, in another study, Zhu et al [22] demonstrated that cancer stem cells in HCC are characterized by the co-expression of CD133 and CD44; these cells also showed preferential expression of some stem cell-associated genes and were more resistant to chemotherapeutic agents due to the up-regulation of several ABC transporters. Conversely, ICC is one of the most difficult intra-abdominal malignancies. Several reports have shown the existence of cholangiocarcinoma stem cells. Markers, such as CD133, CD24, EpCAM and CD44 have been used to isolate cholangiocarcinoma stem cells. Overall, an enhanced expression of these markers in resected specimens of cholangiocarcinoma was associated with malignant potential [23,24]. Lastly, combined hepatocellular-cholangiocarcinoma (HCC-CCA) is <1% of all liver carcinomas. The quintessential type of HCC-CCA usually have high expression of biliary markers (CK7, CK19, and EMA); CD56, c-kit and EpCAM were expressed in varying extents in all HCC-CCA. CD133, EpCAM and vimentin's expression was significantly high in subtypes with HCC-CCA's stem cell features Regarding the clinical outcome, no significant difference has been reported amongeach subtype. The presence of hepatic stem cell markers (CD56, c-kit, CD133, and EpCAM) in varying extents suggests that the histogenesis of combined HCC-CCA could be strongly associated with hepatic stem cells [25]. Many factors such as the patient's general conditions, macroscopic tumor morphology and its histopathologycal features, as well as effective treatment and adjuvant therapies, have been proven to be of prognostic significance. In recent years, along with the understanding of tumor biology and the development of biology techniques, many molecular factors (biomarkers) have been correlated to prognosis. In this review, we will first discuss the possible sources of liver stem cells and their relation with liver cancer development and we will secondly focus on the prognostic significance of clinical and pathological features of HCC.

### HEPATIC PROGENITOR STEM CELLS

Hepatic progenitor stem cells (HPCs) are multipotent stem cells located within the liver's stem cell compartment, the ductal plates of fetal and neonatal livers and canals of Hering in pediatric and adult livers. The compartment represents the anatomic and physiological link between the intralobular canalicular system of hepatocytes and the biliary tree and resides along sites that project starlike from the portal tracts. The phenotypic profile includes epithelial cell adhesion molecule (EpCAM), neural cell adhesion molecule (NCAM), CD133, CXCR4, SOX9, SOX17, FOXA2, cytokeratins (CK) 7/8/18/19, Hedgehog proteins, intranuclear telomerase protein, claudin 3, MDR1, weak expression of albumin and MHC antigens. They do not express alpha-fetoprotein (AFP), intercellular adhesion molecule (ICAM-1), markers for hemopoietic (CD34, CD38, CD45, CD90), endothelial (VEGFr, CD31) or mesenchymal cells (CD146, desmin, CD105) [26]. Conversely, hepatoblasts are diploid bipotent cells from which hepatocytic and cholangiocytic lineages arise. They reside throughout the parenchyma of fetal and neonatal livers or as single cells and small cell aggregates tethered to the ends of the canals of Hering in adult livers [27]. They expand during regenerative processes and have an antigenic profile that overlaps with hepatic stem cells. Protein expression changes include reduction in EpCAM levels, elevated albumin levels switch from NCAM to ICAM-1, expression of CK14 and CK19 and strong positive expression of hepatic specific AFP [28]. At last, in the liver there are “committed precursors” that are unipotent, immature cells. They lose most stem cell gene expression and express either hepatocytic or biliary markers and abound in fetal and neonatal tissues or chronic liver disease [29].

### LIVER CANCER STEM CELLS AND HEPATOCELLULAR CARCINOMA

**Figure 3:**
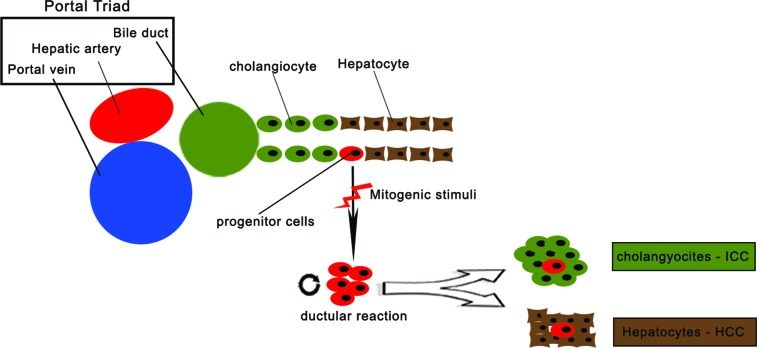
Anatomical location and differentiation capability of hepatic stem cells (adapted from Kruitwagen et al, 2014) Schematic representation of the anatomical location of the hepatic stem cell in the canal of Hering. Upon activation the normally quiescent stem cells proliferate. Depending on the disease, the progenitor cells differentiate into either hepatocytes or chlangiocytes.

Liver cancer is an aggressive disease with a poor outcome. The prevalence of HCC differs greatly by geographical locations. Eastern countries and sub-Saharan African regions have a significantly higher incidence rate than Western countries. Liver cirrhosis caused by viral hepatitis, excessive alcohol consumption and other causes such as Wilson's disease are the major risk factors for HCC development [30]. Despite some improvements in cancer treatment have been made, existing therapies are limited in their ability to cure malignancies and to prevent metastases and relapses. Surgery, radiofrequency, ablation therapy and chemotherapy are all directed at reducing the tumor bulk. In fact, the emergence of CSC theory lends insight into the explanation of why treatment often may be seen initially successful, but eventually results in failure to eradicate the tumor and possibly also in tumor relapse. Similarly with other solid tumors, hepatic CSCs exist and contribute to the development of HCC. Several different markers can be identified such as CD133, CD90, CD44, CD326 (EpCAM) or by selecting the side population (SP) cells by Hoechest dye-staining in order to isolate CSCs from liver cancers. It is important to mention the embryology of the liver to understand the multiple processes of hepatocarcinogenesis. In mammals, the liver bud gives rise to cells destined to become bipotential liver progenitor cells (LPCs) [31]. LPCs initially express alpha fetoprotein and albumin and, later, cytokeratins (CKs)-7 and -19. At this time, cells, called hepatoblasts, positive for CK16 and HepPar1, proliferate, delaminate and invade the septum trasversum mesenchyme (STM) undergoing cellular proliferation and differentiation [32]. Subsequently, LPCs diverge along two cell lineages: hepatocytes (alpha fetoprotein and album positive) and cholangiocytes (CK19 positive). Modulation of biliary and hepatocyte morphogenesis during development is also prominently controlled by TGF-beta signaling via Smad proteins. In adult human tissues, immature epithelial cells have been found residing in the smallest terminal branches of the biliary tree known as the Canals of Hering [33]. These cells have been described as hepatic progenitor cells. Within the niche (Canals of Hering), hepatic progenitor cells are in direct physical continuity with hepatocytes at one membrane boundary and bile duct at another boundary and are considered to represent hepatic stem cells [34]. When the mature epithelial cell compartments of the liver (hepatocytes and/or cholangiocytes) are damaged, a reserve cell compartment is activated [35] (Figure 3). As progenitor cells are activated in most chronic liver diseases that are known risk factors for the development of HCC as well as ICC, progenitor cells are potential target cells for carcinogenesis. The hypothesis that HCC arises from HPCs is supported by the finding that many tumors contain a mixture of mature cells and cells phenotypically similar to HPCs. Most tumors still show phenotypical features of their cell of origin and the histopathological classification is largely based on this. Furthermore, HCCs' immunophenotype revealed the expression of progenitor cell markers such as CK7, CK19, OV6. These tumors, morphologically speaking, consist of cells with a phenotype which is intermediate between progenitors and mature hepatocytes. Especially, CK19 expression in HCC has been associated with a worse prognosis and the expression of CK19 and CK7 in HCC has been associated with a lower tumor free survival rate [36].

### HEPATOCELLULAR CARCINOMA STEM CELL MARKERS

HCC expressing stemness-related markers is a recently suggested subtype of HCC in which a fraction of tumor cells (>5%) expresses stem/progenitor cell markers, not otherwise recognizable by routine analyses stain. This subpopulation of HCCs has been reported to show a more aggressive behavior, compared with conventional HCCs without stemness-related marker expression; therefore, it is important to develop a suitable marker in order to facilitate its diagnosis [37]. Hence, CSCs markers can be considered as useful biomarkers for the HCC's prognostic estimation and eventually for specific targeted therapy. To date, it has been demonstrated that CSCs in HCC can be identified through several cell surface antigens such as CD133, CD90, CD44, OV6 and the epithelial cell adhesion molecule (EpCAM), or by selecting for the side population (SP) cells. In this context, CD133 has been related to a worse prognosis and metastasis in HCC patients. CD133, a transmembrane domain glycoprotein, is an important cell surface marker for both normal stem cells and CSCs in various tissues, including liver. Ma et al [38] proved that CD133 was upregulated in association with the liver regeneration process. Conversely, isolated CD133+ HCC cells were shown to have higher proliferative and tumorigenic potential and to express lower levels of mature hepatocyte markers. Moreover, the expression of CD133+ cells gives to the HCC resistance to conventional chemiotherapy. However, CD133 was found to be expressed in only a minimal proportion of HCC cells, and not expressed at all in normal hepatocytes. This result suggests that the CD133+ cells are responsible of the origin of liver cancer. CD133+ liver CSCs showed activation of the Akt pathway and chemoresistance to doxorubicin or 5-FU. Regarding CD90, its expression on progenitor cells is related to tumor development/progression. This glycosylphosphatidylinositol-anchored conserved surface protein, has emerged as another putative marker for liver CSCs and has been correlated by Yang and colleagues, with tumorigenicity and metastatic potentials in the panel of HCC cell lines that have been tested [39]. In order to exclude lymphocytes, they combined its use with CD45 to isolate non-lymphoid CD90+ cells. They then found these CD90+/CD45− cells to be present in all the HCC cell lines. Furthermore, the CD90+/CD45− cells were present also in blood samples collected from the same HCC patients [40], indicating the presence of putative circulating CSCs. Yang and colleagues performed also multiparametric analyses of CD90+ cells and found that most of them were positive for CD44. This result suggests that the CD90+, CD44+ and CD45− cells are responsible of tumor metastasis. CD44 is a receptor of hyaluronate involved in cell-cell adhesion and migration and it has been associated with tumor cell invasion and migration in liver cancer. In HCC, CD44 is also an important marker used in combination with other CSC markers in order to better define the surface phenotype of liver CSCs. Cells co-expressing CD133 and CD44, or CD90 and CD44 show a more aggressive phenotype. CD133+/CD44+ cells exhibit a more tumorigenic and chemoresistant profile compared to the negative counterpart. Zhu et al [22] showed that CD133+/CD44+ cells possess greater survival when exposed to cytotoxic drugs such as doxorubicin and vincristine. The overexpression of ABC transporters underlies the mechanism, which is responsible of drug resistance. EpCAM (CD326) is a cell surface molecule expressed in almost all the epithelial tumors; it has been identified as a potential early biomarker for HCC [41]. EpCAM is present in the embryonic liver, bile duct epithelium and proliferating bile ductules in the cirrhotic liver, but it is absent in normal adult hepatocytes [42]. Furthermore, EpCAM has been shown to be a direct transcriptional target in the Wnt/β-catenin signaling pathway [43]. This pathway plays an important role in managing the cancer cells' self-renewal. EpCAM has been firstly identified in premalignant hepatic tissue, hence it was suggested to be an early biomarker for HCC. Particularly, the double positivity of EpCAM and AFP in HCC has the worst prognosis [44]. EpCAM+ HCCs are responsible of early relapse after radical resection due to microdissemination in the residual liver because of the high frequency of portal vein invasion [45]. In addition to EpCAM+, CSCs showed chemoresistance against the genotoxic reagent 5-fluorouracil (5-FU). Oval cells (OV6) are one of the most important sources of liver stem cells. These are small cells with a characteristic ovoid nucleus and a underrepresented cytoplasm. They are located in the periportal region and then infiltrate along the bile canaliculi. Oval cells are bipotential and are capable of differentiating into hepatocytes or cholangiocytes [46]. In severe hepatocellular necrosis, chronic viral hepatitis, alcoholic and non alcoholic liver disease, where mature liver cells are unable to regenerate, activation of the potential stem cell compartment leads to formation of reactive ductules with a high expression of oval cell markers. It was demonstrated that OV6+ HCC cells are endowed with a greater tumorigenic ability and chemoresistance to standard chemotherapy. Side population (SP) profile is another method used to identify hepatic CSCs [47]. Stem cells expressing high levels of ATP-binding cassette (ABC) transporters possess the ability to efflux xenobiotic substances. In HCC, these populations are characterized by a higher proliferative potential, antiapoptotic property and higher tumorigenic potential. The use of this method to define liver CSCs has, however, been challenged by some authors disapproving it as the dye used is highly cytotoxic. CD13 (Aminopeptidase N) is a membranous glycoprotein playing an important role in cancer progression. Most recently, CD13 positive cells could be found in the peripheral areas of HCC [48], and this is considered to be related to tumor relapse. Researchers also assessed the tumorigenic potential of CD13+ cells co-expressing CD13 and CD133 or CD90. In fact, CD13 is a marker of tumor-initiating and potentially dormant HCC cells. Studies on CD13 expression and their relationship with the cell-cycle phase indicated that most of the CD13+ fraction exists in the G1/G0 phase. Subsequently, dormant CD13+ cells produce proliferating CD90+ cells that are responsible of metastasis. Immunohistochemical findings also support the view that CD13+ cells play a role in the liver cancer relapse. The apparent increase in the number of CD13+ cells near the fibrous capsule after TACE (transcatheter arterial chemoembolization) is consistent with the fact that clinical HCC relapse after TACE is frequent at the capsule site, due to amplification of CD13+ cells after 5-FU treatment. This suggests that the cellular components of capsule play a role as a protective niche. Cytokeratins are typical epithelial cell markers that are present in many malignant epithelial cells with increases metastatic ability and malignancy [49,50]. It has been reported that several cytokeratin subtypes are also expressed in HCC [51] such as CK19 and CK7. CK19 is a low molecular weight cytokeratin and its correlation with HCC metastasis has been reported [52]. CK19 positive HCCs more frequently showed major vessel invasion and increased tumor size. CK19 expression is therefore considered a significant independent predictive factor of poor disease-free survival [52].

### DIAGNOSTIC AND PROGNOSTIC MARKERS

Hepatocellular carcinoma is one such cancer that can benefit from tumor biomarkers' diagnostic, therapeutic, and prognostic capabilities. The prognosis of patients with HCC still remains dark. The life expectancy of HCC patients is hard to predict. The high rate of intrahepatic and/or extrahepatic post-operative relapse remains one major obstacle to further improving the survival rate and prognosis of HCC patients. Molecular markers used for HCC diagnosis can be classified into three major categories: 1. Traditional serological markers; 2. Cancer stem cell markers; and 3. Tumor tissue markers. Serological markers are most commonly used clinically, whereas among the conventional tumor markers, serum alpha-fetoprotein (AFP) is not only used for diagnosis, but also as a prognostic indicator for HCC patients. AFP is indeed abundantly expressed in fetal liver cells, but not in normal adult liver cells. Patients with high AFP levels at the time of diagnosis tended to have greater tumor size, bilobar involvement, massive or diffuse types and portal vein thrombosis [53]. In order to increase the specificity of the test, some alternative forms of the protein were studied (AFP-L1, AFP-L2 and AFP-L3). The AFP-L3 is the most commonly evaluated isoform in patients with HCC, while AFP-L1 prevails in patients with non-neoplastic disease [54]. Unfortunately, AFP serum concentrations do not correlate well with the prognostic values of HCC such as tumor size, stage, or disease progression. Other serological markers of HCC include Glypican-3 (GP3) that is a member of the heat-shock protein family and plays role in cell growth, differentiation and migration. GP3 promotes the growth of HCC by regulating the signaling activity of several growth factors, including the Wnt/β-catenin pathway, which is crucial for the progression of HCC. In addition, no correlation between GP3 expression and tumor stage, size and AFP level has been observed. Other serological markers of hepatocellular carcinoma include gamma-GT (GGT), and alpha-1-fucosidase (AFU) isoenzymes. None of these markers proved to be more convenient in terms of AFP diagnostics' accuracy. Hepatic Kupffer cells and endothelial cells of the bile duct mainly secrete GGT in healthy adults and its activity increases in HCC tissues. AFU is a lysosomal enzyme found in all mammalian cells. Its activity is found to be increased in the serum of HCC patients. GGT and AFU's measurement is useful in association with AFP in the early diagnosis of HCC and could serve as a valuable supplementary to AFP.

Over the last decade, new technologies (microarray and sequencing) have emerged, leading the search for biomarkers into a new era [55]. Using these technologies, it is now easy to examine a whole tumor genome, transcriptome, epigenome and miRNA profile [56]. Currently, numerous circulating and tissue markers have been identified; however, few biomarkers are acceptable for clinical utility alone for their low predictive accuracy. For the purpose of early diagnosis, HCC can be assessed with serum markers such as AFP level combined with imaging techniques (ultrasonography, magnetic resonance and computer tomography). Tumor tissue-oriented markers are not highly practical because not all tumor tissues can be obtained at an early stage [57], while biomarkers from body fluids such as serum, plasma, urine, and bile are suitable candidates for early diagnosis. This finding led to the conclusion that the serum markers' panel had considerable clinical value for the early diagnosis of HCC and could help patients with an optimal therapy. Surgical treatment, instead, offers a potentially curative option for HCC patients, but outcomes varied due to differing tumor characteristics. The prognosis does not simply reflect the size and number of the tumors but prognosis is affected by a complex interplay of factors, including tumor biology, patient condition, sex, age, and clinicopathological characteristics. Circulating biomarkers such as serum AFP, VEGF, HGF, and TGF-β are still preferred for prognostic prediction. Circulating tumor cells (CTCs) may reflect tumor aggressiveness and serve as a promising candidate for predicting relapse and metastasis [58]. However, their utility is limited by the rarity of CTCs in the peripheral blood even if recent technical findings have made it possible to detect CTCs in multiple tumor types, including breast cancer [59], lung cancer [60] and others [61,62]. Concerning HCC, studies proved that EpCAM+ CTCs may serve as a prognostic marker after curative resection [63]. Research into tumor tissues can provide direct biological information about the tumors. According to the CSC theory, CSCs could influence patient's prognosis by promoting metastasis and recurrence. Consistent with this hypothesis, recent findings show that the presence of CSCs (such as CD90, CD133, CD13, CD44, EpCAM and CK19) could be linked with patient's survival. For instance, the overexpression of CD90 in HCC is associated with poor diagnosis [64]. CD90 is expressed by hepatic stem/progenitor cells during liver development but not in the adult liver. The CD90 positivity has been shown to propagate tumorigenicity. Lu et al [64] performed immunohistochemical studies that confirmed the correlation between CD90 expression and clinical parameters: CD90 was increased in 73% of HCC samples. CD90's overexpression was correlated with age, hepatitis B virus, infection and histological grade, but was not influenced by chronic alcohol exposure or cirrhosis. Therefore, CD90+/CD44+ cells may also serve as a sensitive and specific marker for early tumor diagnosis in HCC. Furthermore, increased CD133 expression is an independent prognostic factor for survival and tumor relapse in patients with HCC [65]. CD133 is a valuable marker expressed in human HCC, while absent in normal liver cells. Sasaki et al [66] shows that cytoplasmic expression of CD133 in HCC is associated with elevated serum AFP levels, histologically high-grade tumor, and tumor invasion to the major branch of the portal vein. Some studies [67,68] demonstrated that CD133 expression is associated with clinical and pathological factors, including preoperative serum AFP level and poorly differentiated tumors. Moreover, a significant association was observed between cytoplasmic expression of CD133 and overall survival of patients with HCC, due to multicentric carcinogenicity and hematogenous metastasis to the liver and remote organs. Consequently, positive cytoplasmic expression of CD133 represented the risk of poor prognosis, especially in patients with HCC at an advanced stage. Particularly, Chan et al [67] showed that CD133 is a highly effective prognostic factor for overall survival in patients affected by disease at stage I. The result of this study not only consolidates the prognostic role of CD133 expression in HCC, but also highlights the significance of CD133 in early stage HCC. EpCAM, conversely, has been associated with younger age, poorer histological differentiation, vascular invasion and more advanced TNM stages [68]. Lastly, CK19 positivity in HCC was well correlated with the clinical and pathological features of tumor aggressiveness and poor prognosis. CK19 positivity in HCC was associated with increased expression of epithelial-mesenchymal transition (EMT)-related genes and invasion-related proteins and these results suggest that this HCC subset may acquire more invasive characteristics, compared to other stemness markers. In the end, CD44's expression in HCC is related to a higher frequency of extra hepatic metastasis and shortened survival rate [69] and CD24's overexpression in HCC correlates with a more aggressive tumor behavior and poor clinical outcomes [70]. However, the predictive range of a single marker is limited to a very small subpopulation, because of the high extent of HCC heterogeneity. Some independent researchers planned a predictive model using a combination of several hepatic CSC's markers in association with clinical and pathological c ha r a c t e ri st i c s [71]. For instance, Yamashita and colleagues [72] have found that AFP could be used with EpCAM to define cancer cells with stem cells features and demonstrated that EpCAM+ and CD90+ cells resided distinctively; furthermore, gene expression's analysis of sorted cells suggested that EpCAM+ cells had features typical of epithelial cells, whereas CD90+ cells had those typical of vascular endothelial cells. Clinical and pathological analysis indicated that the presence of EpCAM+ cells was associated with poorly differentiated morphology and high serum alpha-fetoprotein (AFP), whereas the presence of CD90+ cells was associated with a high incidence of distant organ metastasis. This statement in combination with a serum AFP test might potentially be useful in subgrouping HCC patients and predicting their outcomes. More studies focusing on the identification of other hepatic CSCs population in combination with AFP serum test and clinical-pathological features might significantly improve the current early diagnostic methods.

### NEW POSSIBLE STRATEGIES

The failure of existing cancer treatments has started the search for new methods that effectively target CSCs. The successful eradication of cancer requires anticancer therapy that affects the differentiated cancer cells and the potential CSC population. CSCs are difficult to treat with conventional methods because of their chemoresistant and radioresistant properties, as well as their ability to stimulate angiogenesis and metastasis. Therapies that exclusively address the pool of differentiated cancer cells but fail to eradicate the CSC compartment might ultimately result in relapse and the proliferation of therapy-resistant and more aggressive tumor cells. An ideal drug would kill differentiated cancer cells and, at the same time, selectively kill CSCs to avoid the toxic side effects for other cell types. Novel therapies may be needed in order to overcome the complex network of signaling pathways, and ultimately inhibit the signaling that controls tumor growth and survival. In some review, authors [73,74] have discussed the different therapeutic strategies that could be used in the treatment of liver cancer. CSCs are protected from conventional therapies by changing their microenvironment and self-protection. Specifically targeting any of these areas may lead to the eradication of HCC CSCs. Targeting key signaling pathways for CSC self renewal such as the Wnt/beta-catenin signaling pathway is indeed one approach to therapy. This system/pathway could be inhibited through the development of molecules that destroy the enzymes necessary for the operation of the cascade Wnt/beta-catenin, or through the development of anti-EpCAM antibodies that interfere on its target genes. Another approach is to induce differentiation of the CSCs and then lose their self-renewal properties. In this case, oncostatin M, an interleukin known to induce differentiation of hepatoblasts into hepatocyte, could be used to effectively induce differentiation and cell division of EpCAM+ liver CSCs and the combination of oncostatin M with conventional chemotherapy (5-fluorouracil) could efficiently eliminate HCC by targeting both CSCs and non-CSCs. The recent findings regarding the identification and characterization of liver CSC markers offer great promise for developing better therapeutic strategies against the disease. For example, CD133 cells possess chemoresistance by activation of the AKT/PKB. A possible strategy is to use an AKT1 inhibitor or, in addition, suppression of CD133 by antibody to human CD133 conjugated to a potent cytotoxix drug. Similarly, it would be useful to develop anti-CD44, anti-EpCAM, anti-CD13 antibodies as novel target therapies. The discovery of liver CSCs has also elucidated the mechanisms underlying treatment failure in liver cancer. At present, conventional anticancer therapies including chemotherapy, radiation and immunotherapy, kill rapidly growing differentiated tumor cells, thus reducing the tumor mass, but potentially leaving behind cancer-initiating cells. In addition, microenvironment surrounding them is important for maintenance, such as angiogenesis, vasculogenesis and hypoxia. Many new therapeutic strategies targeting CSCs have been tried (Table 1) [73,74]. Several studies have reported the crucial role of microRNAs (miRNAs) in tumor cell proliferation, apoptosis, metastasis and drug resistance [75]. MiRNAs, a class of non coding RNAs made of 15-25 nucleotides, have been proposed as novel biomarkers in the diagnosis and prognosis stratification of HCC [76]. The differential expression of miRNAs in HCC cells indicates the potential value of miRNA detection in the prediction of HCC diagnosis and prognosis (Table 2). Downregulation of associated HCC miRNAs was significantly related to poor diagnosis, shorter disease-free survival rates and features of metastatic tumors including venous invasion, microsatellite formation and reduced differentiation but also high levels of other types of associated HCC miRNAs has correlated with risk recurrence and shorter overall survival (Table 2). In addition, miRNAs play a pivotal role in the pathogenesis and progression of the disease through the deregulation of various molecular pathways such as PTEN/Akt, p53, RAS/MAPK, Wnt/beta-catenin and others. Other studies [77,78] have suggested that strategies based on the modulation of miRNAs activity may provide a novel approach in the treatment of HCC by inhibition, replacement or regulation of HCC chemosensitivity activities.

### ROLE OF BONE-MARROW STROMAL CELLS OR ADIPOSE STEM CELLS IN LIVER REGENERATION AFTER HEPATIC FAILURE

Liver cirrhosis on the basis of a chronic hepatitis B or C, autoimmune hepatitis, chronic alcohol abuse, primary sclerosis cholangitis and primary biliary cirrhosis are only few of the possible reasons for liver failure. The most common cause of chronic liver disease is chronic liver hepatitis which causes inflammation, necrosis and deposition of collagens at the portal and periportal level, producing damage fibrosis that is the ultimate endpoint. The only effective treatment is orthotopic liver transplantation, accessible only for a limited number of patients, due to lack of donor organs and difficulties with hepatocyte supply. A very encouraging approach lies in regenerative medicine, which holds promise for the development of a cell-based therapy of the liver and may allow the transplantation of hepatocyte-like cells generated from stem cells [79]. Mesenchymal stem cells (MSCs) represent an advantage for allogenic transplantation because they are immunoprivileged with low major histocompatibility complex (MHC) I and no MHC II expression, therefore reducing the risk of rejection and preventing graft-versus-host-disease (GVHD). MSCs can be easily obtained from a the patient's own tissues, isolated ex vivo, expanded and then transplanted into the patient. The most promising regenerative cells are MSCs found in human bone marrow [80] and adipose tissue [81-83]. These stem cells can differentiate in vitro into multiple types of lineages such as: chondrogenic, osteogenic, adipogenic, myogenic, neurogenic and also into hepatogenic lineage.

**Table 1:** Strategies in Eradicate liver cancer stem cells

Blockade of CSC pathways		
Anti-self-renewal	Inhibiting Wnt/β-catenin pathway Suppression of Hedgehog pathway	Oishi N and Wang XW, Int J Biol Sci, 2011
Anti-tumor growth	Anti TGF-β Anti IL-6	Mazzocca A et al., Hepatology, 2009 Barathan M et al., Cell Death Dis, 2013
Anti-survival	4-methylthiobutyl isothiocyanate (MTBITC, erucin)	Herz C et al., J Cell Mol Med, 2014
Differentiation	Hepatocyte nuclear factor-4α Interferon therapy Oncostatin M	Xue TC et al., Oncol Rep, 2014 Zhang W et al., Mol Clin Oncol, 2014 Kong N et al., Asian Pac J Cancer Prev, 2013
Ablation of prospective markers		
Anti-EpCAM	Catumaxomab Adecatumumab	Zhang P et al., Cancer Immunol Immunother,2014
Anti-CD133	AKT1 inhibitor	Smith LM et al., Br J Cancer, 2008
Anti-CD44		Wang L et al., Biomaterials, 2012
Anti-CD13	5-fluorouracil	Haraguchi N et al., J Clin Invest, 2010
Disruption of microenvironment		
Anti-angiogenesis	Activation of the RAF/MEK/ERK Activation of PI3K/AKT/mTOR	Yang L et al., Metallomics, 2014 Chiablaem K et al., Anticancer Res, 2014 Siveen KS et al., Oncotarget, 2014
Anti-vasculogenesis	Vatalanib and Cediranib Bevacizumab Sorafenib, Sunitinib, Brivanib and Linifanib	Katsura et al., Ann Surg Oncol, 2013 Gordon et al., Clin Cancer Res, 2014 Gish RD et al., Clin Adv Hematol Oncol, 2013
Anti-invasion	Metformin Paeoniflorin	Hsieh SC et al., Amino Acids, 2014 Lu JT et al., Bratisl Lek Listy, 2014
Anti-migration	Metformin Glabridin	Hsieh MJ et al., Br J Pharmacol, 2014
Anti-hypoxia	Silencing of hypoxia-inducible factor 1β (HIF-1β)	Choi SH et al., PLoS One, 2014
Disruption of self-protection		
Anti-immune evasion	Depletion of regulatory T cells Myeloid-derived suppressor cells	Sui Q et al., J Immunol, 2014 Hoechst B et al., Hepatology, 2009
Anti-multiple drug resistance	Anti ABCG2	Yan F, Asian Pac J Cancer Pre, 2014
Anti-radioresistance	Enhanced tumour oxygen by recombinant human serum albumin-Fe cyclohexanoil heme	Horinouchi H et al., Cancer Sci, 2008

**Figure 4:**
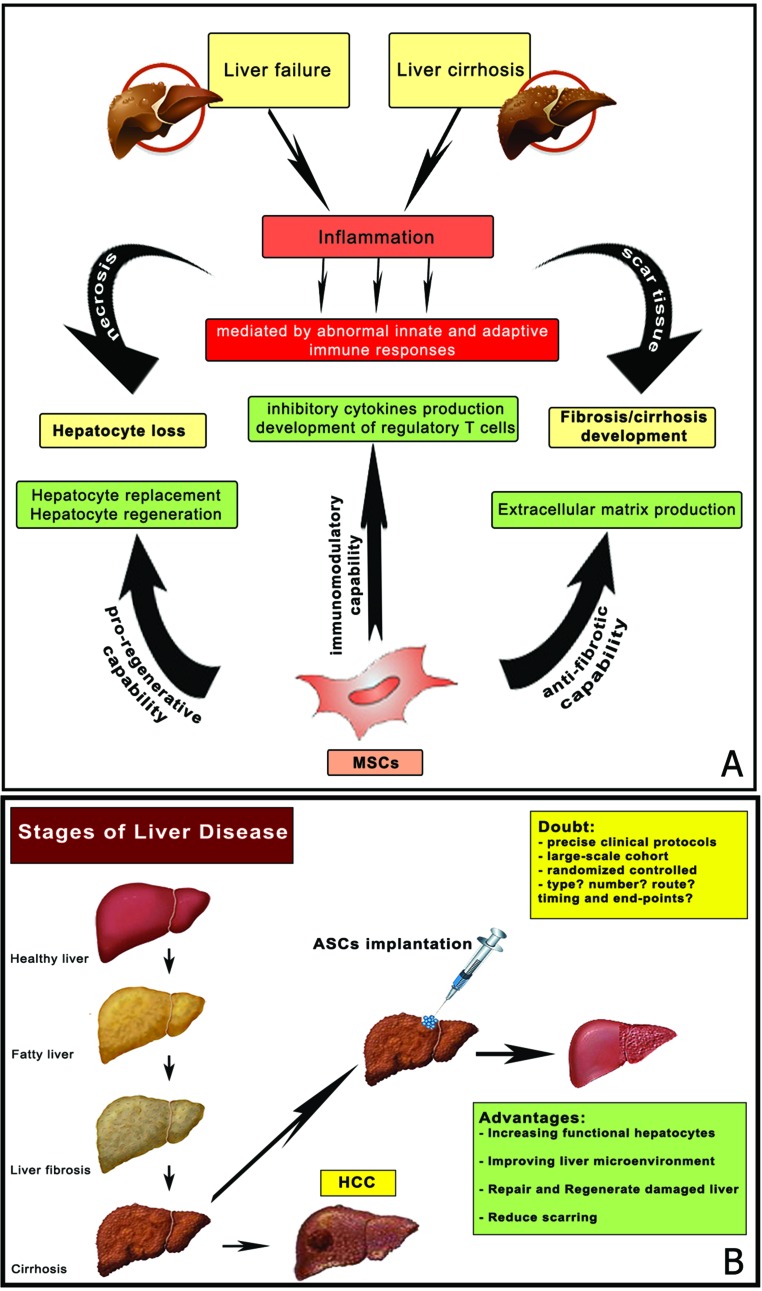
Stem cell therapies for liver failure and cirrhosis (adapted from Zhang Z and Wang FS, 2013) (**A**) Liver failure and cirrhosis are characterized by massive inflammation, necrosis and accumulation of scar tissue. Different stem cell types have various putative functional roles. MSCs are gifted with immunomodulatory, anti-fibrotic and pro-regenerative capabilities. MSCs have been demonstrated to play an immunomodulatory role through producing inhibitory cytokines or inducing the development of regulatory T cells. MSCs have also an anti-fibrotic role for the potential to differentiate into myofibroblasts, which act as scar-forming cells within the liver. The last realistic target of MSC therapy is to replace damaged hepatocytes with exogenous functional hepatocytes in patients with liver failure or cirrhosis. In this regard, MSCs have a pro-regenerative role in fact have been shown to be most capable of producing large numbers of functional hepatocyte-like cells. (**B**) The use of stem cells transplantation may offer novel therapeutic interventions for liver failure and cirrhosis. Several critical issues in clinical protocols require further investigation, such as the optimal type of ASCs, the optimal therapeutic timing, the most effective number of stem cells.

Hence, human adult MSCs are promising candidates for liver regeneration. Multipotent bone-marrow stromal cells (BMSCs) are a group of cells that are found in adult bone marrow. Numerous researchers have focused on the role of BMSCs in the liver regeneration. It is widely accepted that BMSCs differentiate into hepatocytes or hepatic-like cells. In the process of chronic liver disease, BMSCs delay the process of liver cirrhosis and prevent the occurrence of HCC [84]. However, a number of studies have shown that BMSCs have the potential to exacerbate the pathogenesis of tumors and cancer metastasis in situ via cell-cell interaction, secretion of cytokines and growth factors [85]. Therefore, the exact role of BMSCs in cancer development and progression requires further discussion and evaluation before using this stem cells in clinical practice. Thus, many authors focused their attention on the role of adipose stem cells (ASCs). MSC from adipose tissue have been shown to differentiate into hepatocyte-like cells [86]. Transplantation experiments of hepatocytes generated from ASCs into injured nude mice revealed direct incorporation into the liver, which was confirmed by human albumin staining [87]. These findings suggest the possibility of the ASCs' in vivo differentiation caused by a regeneration microenvironment. These results point to a possible alternative future therapy for patients with acute or chronic liver disease: the transplantation of autologous ASCs (Figure 4A,B).

**Table 2:** Circulating miRNAs candidate biomarkers for HCC

miRNA	Dysregulation in plasma	Result	Clinical significance
miR-16	Down	Significant association with HCC, combination with traditional markers improves diagnostic	
miR-17.5p	Up	Significant association with HCC	Poor prognosis, metastasis
miR-21	Up	Elevated in HCC	Poor prognosis
miR-26	Down	Elevated in HCC	Shorter overall survival
miR-124	Down	Elevated in HCC	Early recurrence, metastasis
miR-139	Down	Significant association with HCC	Poor survival
miR-182	Up	Significant association with HCC	Intrahepatic metastasis, poor prognosis
miR-195	Down	Significant association with HCC	Proliferation
miR-199a	Down	Significant association with HCC	Reduced time to recurrence
miR-221	Up	Elevated in HCC, correlates with HCC stage and prognosis	Recurrence, metastatic properties
miR-222	Up	Elevated in HCC	Shorter disease-free survival
miR-224	Up	Elevated in HCC	Promotion of growth, proliferation

## CONCLUDING REMARKS

Research into the molecularbiology of hepatocarcinogenesis has identified several biomarkers that could provide additional information for HCC biology and many of them have been shown to have potential predictive significance. The isolation and culture of hepatic cancer stem cells with stemness properties have an important implication for understanding the molecular mechanisms of liver tumorigenesis. A wide variety of molecular markers have been demonstrated to be excellent diagnostic tools for HCC but it is difficult to characterize HCC with a single biomarker. Thus, signatures of a combination of biomarkers may be more valuable for the diagnosis, staging and prognosis of HCC. Specifically, a correlation of HCC-CSCs phenotype to specific hepatic cancer subtypes and to specific clinical and pathological features has not yet been reported in human liver tumors. Briefly, recent evidence suggests that hepatocellular carcinoma (HCC) is organized by a subset of cells with stem cell features (CSCs) that are considered a pivotal target for eradication of cancer; liver CSCs have been identified through the use of various stem cell markers. In this review, we showed that, in primary HCC, the presence of EpCAM+ cells was associated with poorly differentiated morphology and high serum alpha-fetoprotein (AFP). Since AFP level correlates with the degree of HCC malignancy, these situations suggest that EpCAM tends to be expressed in more malignant HCC tissue, playing a role in HCC progression. Furthermore, studies have demonstrated that CD133's expression was associated with absence of tumor capsule that prevent the spread of tumor cells. This suggests that CD133 tends to be expressed in tumors showing potential for invasion and metastasis. Besides, the presence of CD90+ and CD44+ cells was associated with a high incidence of distant organ metastasis. CD90 expression, again, is not only significantly higher in HCC tissues than in normal adult liver tissue, but it also correlates with a higher histopathologic grade and larger tumors. Hence, it was found that the expression of CD90 is related to the degree of differentiation, suggesting that CD90 is involved in the onset and/or progression of HCC. Postoperative relapse is the main cause of death for HCC patients in the long term, and most recurrences occur within two years after resection. Evidence suggests that CSCs may determine postoperative recurrence and metastasis. Besides, the prognosis of patients with HCC is still dismal. Pathological factors suggesting tumor invasiveness such as venous invasion, presence of satellite nodules, large tumor size, and advanced pTNM stage, are the best-established risk factors for relapse. In order to explore whether the expression of liver CSCs markers correlates with HCC oncogenesis, it is necessary to examine the potential associations between the CSC marker's expression and the following HCC clinical and pathological variables such as age, BMI, serum markers (bilirubin, albumin, ALT, AST, GGT, prothrombin time, AFU), other serum markers (AFP, GP3, growth factors), tumor size, tumor capsule, cirrhosis, and Edmondson and Steiner grade. In addition to the clinical and pathological features, it is necessary to evaluate the correlation between CSCs' markers and the overall survival rate (OS) by calculating the Kaplan-Meier survival curves. Preliminary studies performed by Guo Z et al [88] showed that the survival rate is higher in patients who were negative for CSCs markers. Future studies should aim to verify the insights from the existing literature and broaden them defining the optimal mixture of surface markers in order to identify and isolate HCC-CSCs. Besides, it would be very interesting if future studies aimed to explore how CSCs' markers influence relapse and prognosis in HCC patients.
